# IGF2BP family of RNA-binding proteins regulate innate and adaptive immune responses in cancer cells and tumor microenvironment

**DOI:** 10.3389/fimmu.2023.1224516

**Published:** 2023-07-12

**Authors:** Irina A. Elcheva, Chethana P. Gowda, Daniel Bogush, Svetlana Gornostaeva, Anna Fakhardo, Neil Sheth, Kathleen M. Kokolus, Arati Sharma, Sinisa Dovat, Yasin Uzun, Todd D. Schell, Vladimir S. Spiegelman

**Affiliations:** ^1^Division of Hematology and Oncology, Department of Pediatrics, Pennsylvania State University College of Medicine, Hershey, PA, United States; ^2^Department of Microbiology and Immunology, Pennsylvania State University College of Medicine, Hershey, PA, United States; ^3^Department of Pharmacology, Pennsylvania State University College of Medicine, Hershey, PA, United States; ^4^Division of Neonatology, Department of Pediatrics, Pennsylvania State University College of Medicine, Hershey, PA, United States

**Keywords:** IGF2BP/IMP, RNA-binding protein (RBP), interferon-stimulated genes (ISGs), melanoma, leukemia, anti-PD-1, immunotherapy, cancer stem cell (CSC)

## Abstract

Insulin-like growth factor 2 mRNA-binding proteins (IGF2BP1, IGF2BP2, and IGF2BP3) are a family of RNA-binding proteins that play an essential role in the development and disease by regulating mRNA stability and translation of critical regulators of cell division and metabolism. Genetic and chemical inhibition of these proteins slows down cancer cell proliferation, decreases invasiveness, and prolongs life span in a variety of animal models. The role of RNA-binding proteins in the induction of tissues’ immunogenicity is increasingly recognized, but, the impact of the IGF2BPs family of proteins on the induction of innate and adaptive immune responses in cancer is not fully understood. Here we report that downregulation of IGF2BP1, 2, and 3 expression facilitates the expression of interferon beta-stimulated genes. IGF2BP1 has a greater effect on interferon beta and gamma signaling compared to IGF2BP2 and IGF2BP3 paralogs. We demonstrate that knockdown or knockout of IGF2BP1, 2, and 3 significantly potentiates inhibition of cell growth induced by IFNβ and IFNγ. Mouse melanoma cells with Igf2bp knockouts demonstrate increased expression of MHC I (H-2) and induce intracellular Ifn-γ expression in syngeneic T-lymphocytes *in vitro*. Increased immunogenicity, associated with Igf2bp1 inhibition, “inflames” mouse melanoma tumors microenvironment in SM1/C57BL/6 and SW1/C3H mouse models measured by a two-fold increase of NK cells and tumor-associated myeloid cells. Finally, we demonstrate that the efficiency of anti-PD1 immunotherapy in the mouse melanoma model is significantly more efficient in tumors that lack Igf2bp1 expression. Our retrospective data analysis of immunotherapies in human melanoma patients indicates that high levels of IGF2BP1 and IGF2BP3 are associated with resistance to immunotherapies and poor prognosis. In summary, our study provides evidence of the role of IGF2BP proteins in regulating tumor immunogenicity and establishes those RBPs as immunotherapeutic targets in cancer.

## 1 Introduction

RNA-binding proteins (RBPs) are critical players in maintaining normal cellular homeostasis. The *Homo Sapiens* RNA-protein interactome includes more than 1,300 proteins (1). Despite a large number of identified RBPs, relatively few are involved in cancer development and progression. These RBPs include nuclear RNA splicing factors, RNA-editing and RNA-modification enzymes, nuclear pore-associated proteins facilitating RNA nuclear export, and those that regulate mRNA stability, translation, and degradation (2).

Insulin-like growth factor 2 mRNA-binding proteins (IGF2BP1, IGF2BP2, and IGF2BP3) are a family of cytoplasmic proteins regulating mRNA stability and translation in mammalian cells (3). The expression of all three IGF2BPs peaks at certain stages of embryonic development, but only IGF2BP2 continues to stay active in adult tissues. Abnormal expression of IGF2BP2 and reactivation of IGF2BP1 and 3 are often seen during cancer progression. Depending on the tissue and cellular context, all three members of the IGF2BP family selectively support expression of transcriptional master regulators of cell cycle progression and self-renewal (e.g., *HOXB4*, *MYB*, c-*MYC*, *KRAS*), and metabolic enzymes associated with normal and cancer stem cell physiology (e.g., *ALDH1A1*) (4, 5). The abnormal expression of various RBPs, including IGF2BPs, was documented for various types of cancer including leukemia, colon cancer, and melanoma, which triggered a search for selective small molecule inhibitors (6–22).

Immunotherapies emerge as a promising approach against cancerous tumor cells presenting neoantigens and displaying pro-inflammatory activity (23). As tumor sensing by immune cells relies on the same molecular and cellular mechanisms as the detection of pathogens, the innate immune responses facilitating antimicrobial responses and reaction to cellular stress play an important role in the induction and amplification of T-cell antitumor activity (24).

Innate immunity responses involve a variety of hematopoietic cells cells, including monocytes, macrophages, neutrophils, dendritic cells, natural killers (NKs), and mast cells. Their effector responses such as phagocytosis, antigen presentation and cytokine production, induce T cell activity as a part of adaptive immunity. The key role of T cells in antitumor activity was demonstrated by a positive correlation between the presence of T cells in the tumor microenvironment and patient’s outcome, and by the success of CAR-T therapies against some hematologic malignancies. The success of CAR-T cell therapies in solid tumors, however, is hampered by physical barriers to infiltration and intrinsic immunosuppressive factors. Overcoming those barriers by understanding the relationship between pathways regulating immune responses to pathogens, cell proliferation, and programmed cell death are key for creating novel and improving existing approaches to cancer treatments (25, 26).

The initiation of immune responses occurs via pattern-recognition receptors (PRRs), which recognize the basic pathogen’s structural components such as lipids, lipoproteins, and nucleic acids (DNA and RNA) (27, 28). The activation of nucleic acid sensors and innate immunity signal transduction, is a complex array of molecular interactions and chemical reactions that include protein modifications (e.g., ubiquitination, phosphorylation, palmitoylation etc.) and protein trafficking between cytosolic organelles (e.g., endoplasmic reticulum (ER), endosomes, mitochondria, Golgi apparatus), and nucleus. Intracellular expression of inflammatory cytokines and type I IFN-stimulated genes (ISGs) is a result of IRF3/7 and NF-kB transcription factor activity induced by PRR sensors (29, 30).

In this study, we provide new evidence that IGF2BP1 and its paralogs influence innate and adaptive immune responses in normal and cancer cells. We demonstrate that downregulation of the IGF2BP family of proteins increases sensitivity of human and mouse cells to type I and II IFN stimulation resulting in significantly increased expression of ISGs and a decrease in cell proliferation. Mouse melanoma cells with Igf2bp knockouts demonstrate increased expression of mouse H-2D^b^ and H-2K^b^ MHC I, and the capacity of inducing intracellular Ifn-γ expression in syngeneic, large T antigen (Tag)-specific T-lymphocytes *in vitro*. Igf2bp1 genetic inhibition increased tumor immunogenicity, which is in line with a recently published study (20). Finally, we demonstrate that the efficiency of anti-PD1 immunotherapy in the mouse melanoma model is significantly more efficient in tumors that lack Igf2bp1 expression. Analysis of data from human melanoma patients indicates that high levels of IGF2BP1 and IGF2BP3 are associated with resistance to immunotherapies and poor survival. In summary, our study demonstrates that the IGF2BP family of RNA-binding proteins regulate innate and adaptive immune responses in cancer cells and tumor microenvironment, and positions those RBPs as possible immunotherapeutic targets in cancer.

## 2 Materials and methods

### 2.1 Cell lines and cultures

Human embryonic kidney T-large antigen transformed cells (HEK293T), and human melanoma SK-Mel28 were obtained from ATCC. Human melanoma cell line MEL928 was kindly provided by Dr. Paul Robbins (Center of Cancer Research, National Cancer Institute, Bethesda, MD). BrafV600E-driven mouse melanoma cell line, SM1, syngeneic to fully immunocompetent C57BL/6 mice was developed in Dr. Antoni Ribas lab (31) and kindly provided by Dr. Jianxun Song (Texas A&M School of Medicine). The Penn Vet Mouse Melanoma (PVMM) BrafCA; Tyr-CreERT2; Ifnar1^−/−^ cell line was kindly provided by Serge Y. Fuchs (32). Mouse melanoma cell line SW1, derived from a K-1735p lung metastasis, was a generous gift from Dr. Ze’ev Ronai of Sanford Burnham Prebys Medical Discovery Institute (33). HEK293T, SK-Mel28, MEL928, SM1, and SW1 cell lines were maintained in DMEM-high glucose, supplemented with 10% fetal bovine serum and 1% penicillin-streptomycin. The human and mouse cell cultures were routinely tested for mycoplasma using the MycoAlert PLUS Mycoplasma Detection Kit (Lonza, Walkersville, MD).

### 2.2 Establishing murine melanoma B16.F10 cells expressing T large antigen (B16.F10-Tag)

Mouse melanoma cell line B16.F10 was obtained from ATCC. B16.F10-Tag were generated in TS’s laboratory, Penn State College of Medicine. Cells were transfected with plasmid pPVUO-neo expressing large and small T antigens (34). Expression of large T antigen was confirmed with immunostaining. B16.F10-Tag cells were cultured in DMEM-high glucose, supplemented with 5% fetal bovine serum, 1% penicillin-streptomycin and 0.8-1.0 mg/ml of Geneticin (G418).

### 2.3 Generation of human and mouse IGF2BP gene’s knockdown and knockout cell lines

Lenti-viral vectors for short hairpin RNA (shRNA) expression against IGF2BP1, 2 and 3 and non-targeting control were purchased from Sigma (St. Louis, MO). Virus production and transfections were carried out as previously described (35). The selection doses of puromycin were assessed for each cell line and puromycin selection of cells expressing lentivectors was carried out for a week, from day 2 to day 9 days post-transduction. CRISPR/Cas9 plasmids for human and mouse IGF2BP genes knockouts and corresponding non-targeting control were purchased from Santa Cruz Biotechnology Inc., (Santa Cruz, CA) as well as the transfection reagent. Mouse and human cells were transfected with corresponding CRISPR/Cas9 plasmids according to the manufacturer’s recommendation. GFP positive cells were sorted using fluorescence activated cell sorting (FACS) equipment at the Pennsylvania State University, College of Medicine core facility (Hershey), and single GFP positive cells were deposited into 96-well plate for the following single cell clonal selection and derivation of gene knockout and control clones. The experimental conditions for mouse B16.F10-Tag melanoma CRISPR/Cas9 non-targeting control, Igf2bp1, 2, and 3 gene knockout were the same, but Igf2bp1-KO clones were short-lived and not suitable for experiments. Two Igf2bp1-KO clones were successfully generated from SM1 mouse melanoma that were used in this study together with B16.F10-Tag Igf2bp2-KO and Igf2bp3-KO clones, and corresponding SM1 and B16.F10.Tag non-targeting CRISPR/Cas9 controls. A low passage of cells with Igf2bp1-3 gene knockout were used for each biological replicate and the *in vivo* experiments, where Igf2bp1, 2, and 3 protein expression levels were routinely assessed by western blotting compared to corresponding non-targeting shRNA or gRNA controls (**Supplementary Materials and Methods Table 2.1**).

### 2.4 Cytokines treatments

Human interferon beta, gamma, and TNF-α were purchased from PeproTech Inc. (NJ, USA). Murine interferon-gamma was obtained from PeproTech Inc. (NJ, USA), and mouse interferon beta was purchased from PBL Assay Science (12405-1). Cytokines reconstitution was handled according to the manufacturer’s recommendations. Briefly, the lyophilized powder was reconstituted in water to a concentration of 1mg/mL, then further in 0.2% BSA to a final concentration of carrier 0.1% BSA and cytokines to 0.5mg/mL or up to 2.5x10^6^ units/mL. Human and murine recombinant cytokines were routinely applied at concentrations: hrIFN-β (10 ng/mL), hrIFN-γ (100ng/mL), hrTNF-α (10ng/mL), mrIfn-β (1000U/mL), mrIfn-γ (50 or 100U/mL). For gene expression analysis, cytokines were added into a standard growth media and incubated for 12 hours prior to the collection of an experiment.

### 2.5 IncuCyte cell growth assay

Human and mouse melanoma cells were seeded at a density 1.5-3.0 x 10^3^ per well, supplemented with either regular growth media or cytokines. At least three biological replicates were performed for each experiment, with three to four technical replicates per sample, at least four area scans per well. The results were analyzed using IncuCyteS3 software.

### 2.6 RNA-Seq sample processing

RNA was extracted using the RNeasy Mini Kit (Qiagen, Germantown, MD), followed by DNase I treatment with DNA-free DNA Removal Kit (Invitrogen, Carlsbad, CA). cDNA was made with the iScript cDNA Synthesis Kit, and quantitative PCR (qPCR) was performed using iTaq Universal SYBR Green Supermix on Bio-Rad C1000 Touch Thermal Cycler CFX96 Real Time System (Bio-Rad, Hercules, CA). qPCR reactions were assembled with two technical replicates, and two to three biological replicates were performed for each experiment. Primer’s sequences are provided in the **Supplementary Materials and Methods Tables 2.2**, **2.3**.

RNA-seq libraries were prepared in the Penn State College of Medicine Genome Sciences core (RRID : SCR_021123) using the Illumina Stranded mRNA Prep, Ligation kit (Illumina) as per the manufacturer’s instructions. Briefly, polyA RNA was purified from 200 ng of total RNA using oligo (dT) beads. The extracted mRNA fraction was subjected to fragmentation, reverse transcription, end repair, 3’– end adenylation, and adaptor ligation, followed by PCR amplification and magnetic bead purification (Omega Bio-Tek). The unique dual index sequences (IDT^®^ for Illumina RNA UD Indexes Set B, Ligation, Illumina) were incorporated in the adaptors for multiplexed high-throughput sequencing. The final product was assessed for its size distribution and concentration using BioAnalyzer High Sensitivity DNA Kit (Agilent Technologies). The libraries were pooled and sequenced on Illumina NovaSeq 6000 (Illumina), to get on average 25 million, paired end 50 bp reads, according to the manufacturer’s instructions.

### 2.7 Gene expression analysis

The paired sequencing reads were aligned to the GRCh38 (hg38, PMID: 28396521) genomic assembly obtained from UCSC Goldenpath (PMID: 12434005), using STAR aligner (PMID: 23104886). Reads with ambiguous alignments (mapping to multiple genomic regions) were filtered out from the analysis. The uniquely mapped reads were quantified across the genes using featureCounts function in R Subread package (PMID: 30783653) using Gencode genomic annotation v38 (PMID: 30357393) for hg38. The normalized gene expression (FPKM: fragments per kilobase of exon per million mapped fragments) were also computed using aligned reads with Cufflinks (PMID: 22383036). Differential expression analysis (DEA) was performed with the quasi-likelihood negative binomial generalized log-linear model (*glmQLFTest*) function in edgeR package (PMID: 27508061) after TMM (trimmed mean of M values) normalization (PMID: 20196867) using raw counts. Only genes that are expressed in at least two samples (FPKM>1) in either group were considered for DEA and statistically significant genes were extracted using the false discovery rate (FDR) threshold of 0.05 and minimum fold change (FC) threshold of 1.5 in either direction. The gene biotype and description information were retrieved via biomaRt (PMID: 19617889). In addition, differentially expressed genes were utilized for gene ontology (GO) enrichment annotation with cluster Profiler package in R and KEGG pathway analysis with STRING (http://string-db.org/). Venn diagrams were generated using R *venn* package (*a*). The heatmaps were generated using R *heatmap* package (*b*). Gene set enrichment analysis was performed by GSEA software using Ratio_of_Classes as the comparison metric using gene sets obtained from Molecular Signature Database (MSIGDB) (PMID: 16199517). Gene sets in the MSIGDB were curated from associated studies (GOBP_RESPONSE_TO_TYPE_I_INTERFERON - PMIDs:15546383, 6681834; REACTOME_INTERFERON_GAMMA_SIGNALING-PMIDs: 34788843, 14525967, 9143700, 18929502, 9462485). (*a*) Adrian Dusa. venn: Draw Venn Diagrams. CRAN, https://cran.r-project.org/web/packages/venn/index.html, 2022. (*b*) Raivo Kolde. heatmap: Pretty Heatmaps. CRAN, https://cran.r-project.org/web/packages/pheatmap/index.html, 2019.

### 2.8 *In vivo* experiments

All procedures were carried out in accordance with the Institutional Animal Care and Use Committee (IACUC) guidelines. Terminally sick mice were euthanized at humane endpoints per IACUC protocol guidelines. Six to eight-week-old males and females C3H mice were purchased from the Jackson Laboratory. To investigate a combined effect of anti-PD-1 antibody, IL-2 and Igf2bp1-KD on tumor formation, groups of five C3H mice were injected into the right flank of mice, subcutaneously, with 1.0 × 10^6^ SW1 cells expressing doxycycline-inducible Igf2bp1 gene knockdown (KD) or non-targeting shRNA control vectors. Five days after injection, Igf2bp1 knockdown was induced *in vivo* by administration of doxycycline-enriched food pellets. Doxycycline treatment was maintained throughout the length of the experiment. *In vivo* tumor formation experiments were randomized based on mice gender and performed as follows: IL-2 (Proleukin) at 120,000 IU twice/day by intraperitoneal injections (i.p.) on days 1-5, 8-12; anti-PD-1 Ab (clone RMP-1, Leinco Technologies, Inc., MO) at 200 μg, i.p. on days 1, 4, 8, 11, 15, 18; control treatment group(s) was treated with i.p. injections of phosphate-buffered saline (PBS). Tumor size was measured three times a week, and tumor volume was calculated based on the following formula: volume (mm^3^) = (length × width^2^)/2. For *in vivo* survival study, forty C57BL/6 (Jackson Laboratory) six-weeks-old *Mus. musculus* males and females were purchased from the Jackson Laboratory. Groups of 10 mice, both genders, were injected subcutaneously into the right flank of mice with 0.5 × 10^6^ SM1 cells with either CRISPR/Cas9 induced Igf2bp1-KO or gRNA non-targeting control. Once tumor palpated, anti-PD1 antibody (described above) or PBS was intraperitoneally injected into mice twice a week for three weeks. Tumor measurements and survival of mice were recorded and analyzed as previously described.

### 2.9 Flow cytometry and tumor microenvironment analysis

C57BL/6 and C3H mice of both genders were injected with 0.5 x10^6^-0.7×10^6^ SM-1 or SW-1 cells correspondingly. Tumors were chopped and resuspended in a collagenase-DNAse cocktail (1 mg/mL of collagenase and 50U/mL of DNAse in RPMI supplemented with 2% FBS). Digestion was performed for 40 minutes, 37°C after which tissues were processed through a 70 µm mesh screen to prepare single-cell suspensions. Red blood cells were lysed using RBC Lysis buffer (BioLegend). Remaining cells were counted and 1×10^6^ cells were used for staining with the lymphoid or myeloid surface markers panel using a standard flow cytometry protocol. Briefly, nonspecific binding was blocked with purified anti-CD16/32 antibodies and cells were stained with Fixable Viability Stain 780 for 15 minutes in the dark. Cell surface staining was performed at room temperature, in the dark for 15-20 min with antibodies listed in **Supplementary Methods Table 2.4**. After incubation with antibodies, cells were washed several times and fixed in 2% paraformaldehyde (PF) for 20 minutes at 4°C. Flow cytometric analysis of H-2K^b^ and H-2D^b^ was assessed using monoclonal antibodies Y-3 and B22.249, respectively, followed by detection with goat anti-mouse IgG-FITC. Intracellular cytokine staining of Ifn-γ was performed by mixing mouse CD8^+^ T cell clone K-11, specific for the H-2D^b^-restricted Tag I 206-215 determinant (36), at a 1:1 ratio with 3x10^5^ tumor cells for 5 hours in the presence of 1 μg/ml brefeldin A. Cells were subsequently stained for surface CD8 and then intracellular Ifn-γ using the Cytofix/Cytoperm (BD Biosciences) kit per the manufacturer’s instructions. Sample acquisition was performed using a FACSymphony A3 flow cytometer (Beckton Dickinson) and data analyzed with FlowJo software. Information about antibodies is provided in the **Supplementary Materials and Methods Table 2.4**.

### 2.10 Statistical analysis

Data were analyzed using GraphPad Prism (GraphPad Software Inc., San Diego, CA, USA) and R programming language version 3.4.4 (R Foundation, Vienna, Austria). A log-rank (Mantel-Cox) test was used to determine p values in Kaplan-Meier survival curves comparison. One-way ANOVA models were used to analyze the differences between 3 or more groups. For two-group analysis, two-sample Student’s, or Welch’s t-tests were used. Tests were two-sided, and values with *p<0.05, **p<0.01, ***p<0.001, ****p<0.0001were considered statistically significant.

## 3 Results

### 3.1 Downregulation of the IGF2BP family of proteins stimulates expression of genes involved in response to pathogens and pro-inflammatory pathways

**Figure 1:**
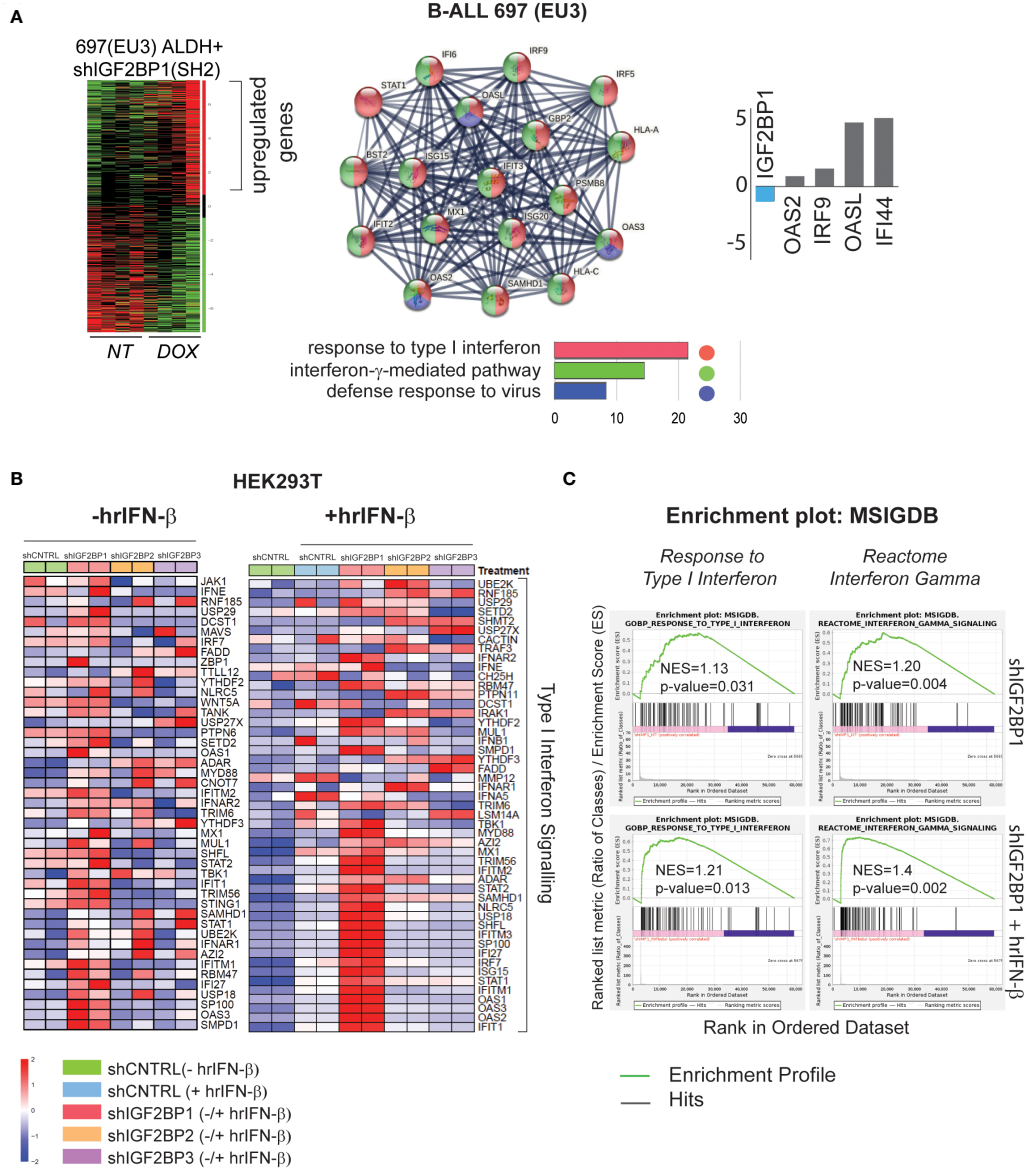
IGF2BP1 regulates type – I and type-II IFN-stimulated gene expression in mammalian cells **(A)** RNA-seq gene ontology (GO) and KEGG pathway analysis, by STRING software, of human B-ALL 697 (EU3) ALDH^+^ cells with a doxycycline-inducible IGF2BP1 knockdown; log2(fold) expression of several interferon-stimulated genes from B-ALL mRNA-seq data set; **(B)** HEK293T cells mRNA-seq analysis: a heat map of differentially expressed, type I interferon signaling genes in HEK293T cells expressing short-hairpin (sh) RNAs targeting IGF2BP messengers (shIGF2BP1, 2, 3) or non-targeting shRNA control (shControl, shCNTRL) before (left map), and after hrIFN-β (10ng/mL, 12 hours (hrs)) treatment (right map). **(C)** GSEA type I interferon pathway (left panel), interferon gamma signaling reactome (right panel) in unstimulated HEK293T cells expressing shIGF2BP1 (shIGF2BP1) or hrIFN-β-treated shIGF2BP1 (shIGF2BP1+hrIFN-β), versus to non-targeting corresponding (unstimulated and hrIFN-β-treated) shControls.

Our previous study of leukemia stem cells (LSC) properties (e.g., tumorigenicity, clonogenicity, and expression of aldehyde dehydrogenase (ALDH) by ALDEFLUOR™ analysis) established IGF2BP1 as an important regulator of the leukemia stem cell phenotype. The gene expression analysis of ALDH-enriched B-ALL 697 (EU3) cell line with IGF2BP1 gene knockdown revealed that somatic stem cell division, ER-nucleus signaling, translation, RNA modification, and cellular response to stress were among most downregulated pathways (based on 1,234 downregulated genes (*p<0.05, FDR<0.3); Gene Ontology (GO) confidence interval 98%, FDR<0.05) (GSE138704) (4). The Gene Ontology enrichment analysis of upregulated genes in the same data set (~1,300 genes) indicates that IGF2BP1 knockdown in ALDH-enriched B-ALL 697 (EU3) cell line induces type I interferon, interferon-γ-mediated, and defense response to virus pathways (**Figure 1A**). Among the most up-regulated genes are interferon induced protein 44 (*IFI44*, 25-fold), 2’-5’-Oligoadenylate Synthetase Like (*OASL*, 20-fold), Toll Like Receptor 10 (*TLR10*, 6-fold), interferon Regulatory Factor 9 (*IRF9*, 2-fold). Importantly, *STAT1* and several *HLA* genes were upregulated as well (**Supplementary Data Table 1.1**).

To further investigate the role of IGF2BP1 and its two paralogs, IGF2BP2 and IGF2BP3, in regulation of innate immune responses, we created shRNA-mediated gene knockdown (KD) and CRISPR/Cas9-based IGF2BP1-3 gene knockout (KO) loss-of-function systems in HEK293T cells (**Supplementary Figures 1A, B**). Induction of interferon-stimulated genes (ISG) by human recombinant interferon beta (hrIFN-β, 10ng/mL, 20ng/mL, 12 hours) was validated by qRT-PCR and western blotting for IFI44 (**Supplementary Figure 1C**). To study the effect of IGF2BP proteins expression on interferon type I signaling, gene expression profiles from PBS-treated HEK293T cells expressing shIGF2BP1, 2 and 3 were compared to that of shRNA non-targeting control samples treated with hrIFN-β. We found that among the three paralogs, IGF2BP1 has a significant effect on interferon beta and gamma signalings gene expression. Downregulation of IGF2BP1 leads to increased expression in 40 out of 176 genes of innate immunity pathway induced by hrIFN-β cytokine in HEK293T cells (**Supplementary Figure 2A**). Upon hrIFN-β stimulation (hrIFN-β, 10ng/mL, 12 hours), interferon beta-induced gene expression and interferon gamma-associated signaling were significantly increased in IGF2BP1 knockouts (**Figures 1B, C**). Relative normalized gene expression of selected interferon-stimulated genes, *IFI44, OAS1, OASL2, RIG-1, STAT1, IRF7*, in HEK293T cells treated with hrIFN-β was significantly increased for most targets in IGF2BP1-3 gene knockdown and knockout, compared with corresponding controls (**Supplementary Figures 2B, C**). Therefore, we conclude that high expression levels of cytoplasmic IGF2BP1 protein negatively affect mRNA levels of interferon type I-stimulated genes in mammalian cells with and without cytokine stimulation. Downregulation of IGF2BP1 and its two paralogs facilitates expression of genes involved in response to pathogens upon interferon stimulation.

### 3.2 Downregulation of IGF2BP family of proteins supports IFN-mediated growth inhibition in melanoma cells

**Figure 2:**
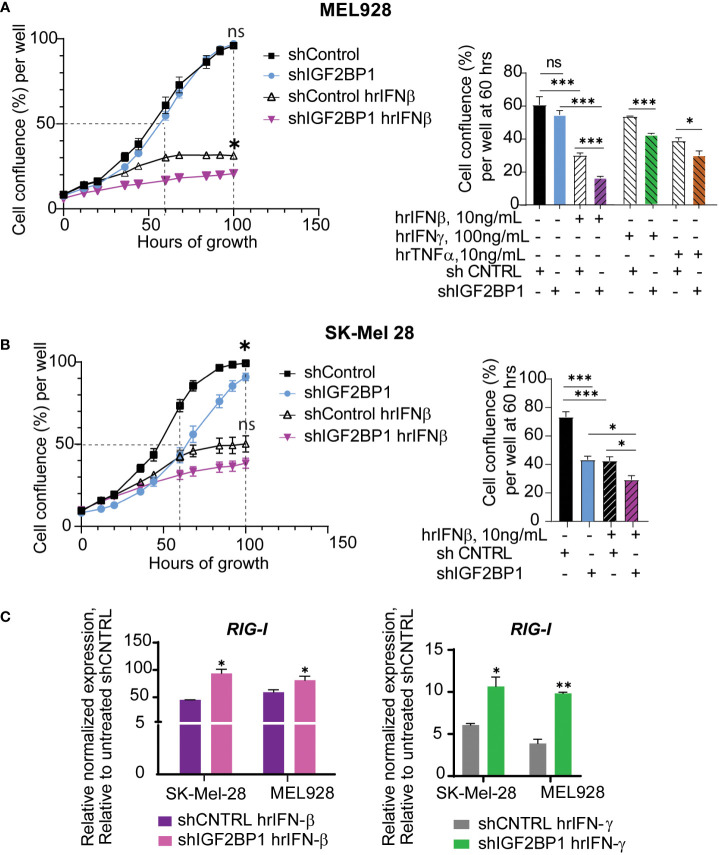
IGF2BP1 downregulation sensitizes human cancer cells to IFN-beta and IFN-gamma treatment. **(A)** IncuCyte™ cell imaging analysis of human melanoma Mel928 cells growth expressing shIGF2BP1 or shControl, stimulated with hrIFN-β (10ng/mL), hrIFN-γ (100ng/mL), and hrTNF-α (10ng/mL) and **(B)** SK-Mel-28 treated with hrIFN-β (10ng/mL); the summary of the unpaired t-tests at 60-hours of growth, approximately 50% cell confluency for most samples, is presented on right graphs; the summary of the unpaired t-tests at 100-hours of growth, the end time point at maximum cell confluency, is presented on the plots (left); **(C)** qPCR for *RIG-1* expression in SK-Mel-28 and MEL928 cells, expressing shIGF2BP1 or shControl, treated with hrIFN-β (left graph), and hrIFN-γ (right graph), relative to unstimulated shRNA control. *p<0.05, **p<0.01, ***p<0.001; not significant (ns, p>0.05).

Taking into consideration the success of immunotherapies in melanoma treatment, we went on to investigate the effect of IGF2BPs expression levels on innate immunity responses in human and mouse melanoma cells using *in vitro* and *in vivo* assays. First, growth of human melanoma cell lines MEL928 and SK-Mel-28 expressing shIGF2BP1 or non-targeting shControl with and without cytokine treatments was assessed with IncuCyte™ technology. Human recombinant interferon beta (hrIFN-β) treatment had a strong inhibitory effect on MEL928 melanoma cell growth compared to treatments with hrIFN-γ and TNFα (**Figure 2A**). MEL928 and SK-Mel-28 cells expressing non-targeting shRNA controls were severely inhibited by exposure to hrIFN-β. The hrIFN-β treatment combined with IGF2BP1 gene knockdown resulted in additional 10-13% growth reduction in both cell lines (**Figures 2A, B**). qPCR analysis of ISGs in MEL928 and SK-Mel-28, expressing shIGF2BP1 or non-targeting shControl and treated with hrIFN-β or hrIFN-γ, showed a consistent upregulation of *RIG-1* expression in both experimental conditions (**Figure 2C**).

**Figure 3:**
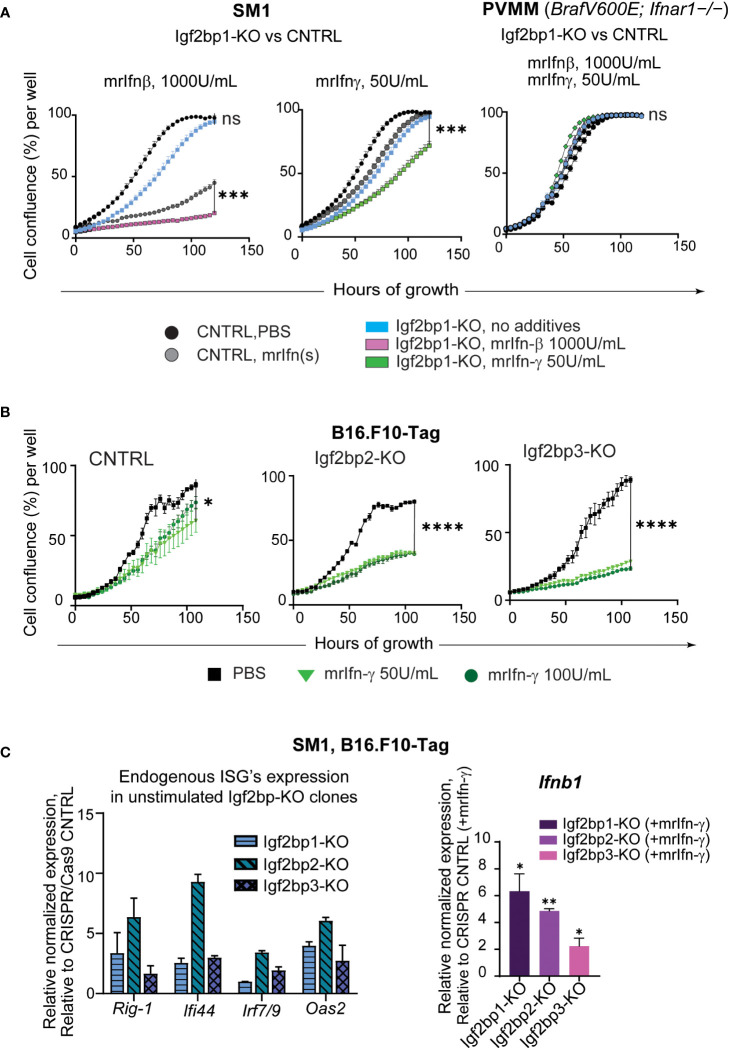
IGF2BPs downregulation sensitizes mouse cancer cells to IFN-beta and IFN-gamma treatment. **(A)** IncuCyte™ cell growth imaging analysis of SM1 mouse melanoma Igf2bp1-KO or non-targeting guide RNA (gRNA) CRISPR/Cas9 control (CNTRL), stimulated with mouse recombinant (mr) Ifn-β (1000Units/mL, left graph) or mrIfn-γ (50Units/mL, center graph). IncuCyte™ cell growth imaging analysis of PVMM (*Ifnar1^−/−^*) cell line Igf2bp1-KO or CRISPR/Cas9 control, stimulated with mrIfn-β (1000Units/mL) or mrIfn-γ (50Units/mL);the summaries of the unpaired t-tests at 120-hours of growth, the end time point at maximum cell confluency, are presented on the plots; **(B)** IncuCyte™ cell growth imaging analysis of mouse melanoma B16.F10-Tag cells genetically edited by CRISPR/Cas9 non-targeting gRNA (CNTRL), gRNA against Igf2bp2 (Igf2bp2-KO), Igf2bp3 (Igf2bp3-KO), and exposed to PBS (unstimulated control), or mrIfn-γ 50U/mL, or mrIfn--γ 100U/mL; the summaries of the unpaired t-tests at 120-hours of growth, the end time point at maximum cell confluency, are presented on the plots; **(C)** Endogenous expression of interferon-stimulated genes (ISGs) in unstimulated mouse Igf2bp1-3-KO clones relative to CRISPR/Cas9 non-targeting control assessed by qPCR (left); Normalized relative expression of endogenous interferon beta (*Infb1*) gene in mouse melanoma cells with CRISPR/Cas9-induced Igf2bp1, 2, and 3 knockouts relative to CRISPR/Cas9 gRNA non-targeting control after mrIfn-γ stimulation (50U/mL, 12 hrs), relative to mrIfn-γ stimulated CRISPR/Cas9 CNTRL. *p<0.05, **p<0.01, ***p<0.001, ****p<0.0001; not significant (ns, p>0.05).

To further investigate the physiological significance of IGF2BPs proteins in innate and adaptive immune responses in melanoma, we created Igf2bp1, 2, and 3 genes knockouts (-KO) in mouse melanoma cell line SM1 and mouse melanoma B16.F10 cells expressing T antigen (B16.F10-Tag) (**Supplementary Figures 3A–D**). Similar to human melanoma cell line MEL928, proliferation of mouse SM1 cells was severely impaired by exposure to mouse recombinant interferon beta (mrIfn-β), and almost abolished after mrIfn-β treatment in Igf2bp1 deficient cells (**Figure 3A**, left graph). Proliferation was also significantly reduced in SM1 shIgf2bp1 cells treated with mrIfn-γ (**Figure 3A**, central graph). The inhibitory effect of both mrIfn-β and mrIfn-γ in Igf2bp1 gene knockout cells was rescued by *Ifnar1* knockout in melanoma cell line PVMM, which shares a genetic background with the wild type SM1 cell line (**Figure 3A**, right; **Supplementary Figures 3E, F**). Genetic knockout of Igf2bp2 and Igf2bp3 exhibited a significant inhibitory effect on B16.F10-Tag cell growth compared to cells expressing CRISPR/Cas9 non-targeting control upon mrIfn-γ stimulation (**Figure 3B**). qPCR analysis of endogenous gene expression in mouse melanoma cells with *Imp1-3* gene knockouts (no cytokine’s treatment) showed an increase of ISGs levels (e.g., *Rig-1, Ifi44, Irf7/9, Oas2*). After 12 hours of stimulation with mrIfn-γ, mouse melanoma clones with Ig2bp1-3 gene knockout expressed significantly higher levels of endogenous interferon beta compared to CRISPR/Cas9 non-targeting controls (**Figure 3C**). Therefore, these data support our previous observation that downregulation of the IGF2BP family of proteins stimulates pro-inflammatory pathways in mammalian cells. IGF2BP downregulation increases sensitivity to interferon stimulation, enhancing interferon-mediated inhibition of mouse and human melanoma cell growth.

### 3.3 Downregulation of IGF2BP family of proteins increases immunogenicity of mouse melanoma cells and inflames tumor microenvironment

**Figure 4:**
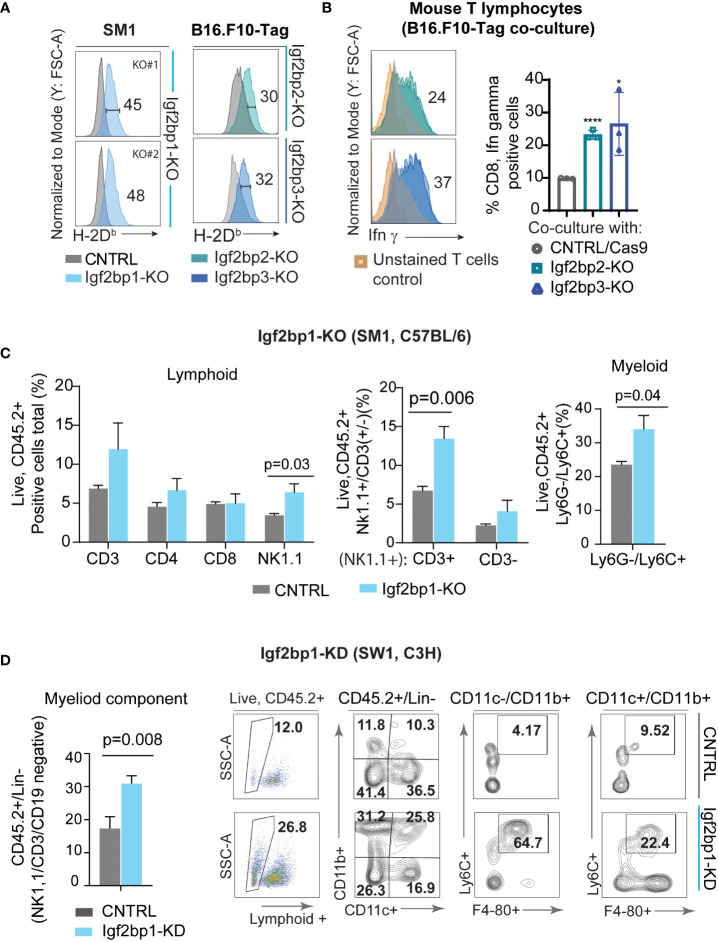
Downregulation of IGF2BP family of proteins increases immunogenicity of mouse melanoma cells and inflames tumor microenvironment. **(A)** Flow cytometric analysis of mouse H-2D^b^ surface expression in SM1 Igf2bp1-KO and B16.F10-Tag Igf2bp2-KO, Igf2bp3-KO cells compared to CRISPR/Cas9 control; *p<0.05, ****p<0.0001; **(B)** flow cytometric analysis of intracellular Ifn-γ expression in mouse CD8+ T-cell clone K-11, co-cultured for 5 hours with B16.F10-Tag Igf2bp2-KO, Igf2bp3-KO, or CRISPR/Cas9 CNTRL clones (*n*=3); **(C)** flow cytometric analysis of mouse tumor microenvironment in SM1 mouse melanoma tumors formed by Igf2bp1-KO or CRISPR/Cas9 CNTRL cells in syngeneic C57BL/6 mice (*n*=6); **(D)** quantification and representative flow cytometry plots for myeloid cells in doxycycline-inducible SW1 Igf2bp1-KD compared to SW1 shControl tumors in syngeneic C3H mice (*n*=7).

Genetic inhibition of Igf2bp paralogs in SM1 and B16.F10-Tag mouse melanoma cells is associated with increased surface presentation of mouse H-2D^b^ and H-2K^b^ complexes detected by flow cytometry (**Figure 4A**; **Supplementary Figure 4A**). Subsequent co-culture of Igf2bp2, 3-KO B16.F10-Tag cells with Tag-specific mouse T lymphocytes, was found to increase intracellular production of Ifn-γ in T cells on average twice as much as compared to control (**Figure 4B**; **Supplementary Figure 4B**). Flow cytometric analysis of myeloid and lymphoid cells extracted from C57BL/6 mouse melanoma model using SM1 Igf2bp1-KO and CRISPR/Cas9 control showed increased accumulation of some immune cell subsets in the tumor microenvironment. The population of CD45.2+/NK1.1+ cells was significantly increased, mainly due to an increase within the CD3+NK1.1+ subset compared to non-targeting control tumors (**Figure 4C**). We also observed an increase in the CD45.2+/Ly6G-/Ly6C+ myeloid subset in the tumor microenvironment of SM1/Igf2bp1-KO melanoma mouse model (**Figure 4C**, right graph). Accumulation of immune cell subsets was also observed in the C3H mouse model using SW1 cell line with Igf2bp1 doxycycline-inducible gene knockdown (**Supplementary Figure 4C**). Myeloid cell accumulation in the tumor microenvironment and skewing toward a macrophage phenotype was increased in Igf2bp1-KD tumors (**Figure 4D**). These results indicate that downregulation of Igf2bp1, 2 and 3 paralogs increases the immunoreactivity of mouse melanoma cells assessed by increased H-2D^b^ expression and antigen presentation to T cells *in vitro*. Genetic inhibition of Igf2bp1 provokes accumulation of lymphoid and myeloid cells in the tumor microenvironment in mouse melanoma models.

### 3.4 Downregulation of IGF2BP1 increases sensitivity to immunotherapy in mouse and human melanoma

**Figure 5:**
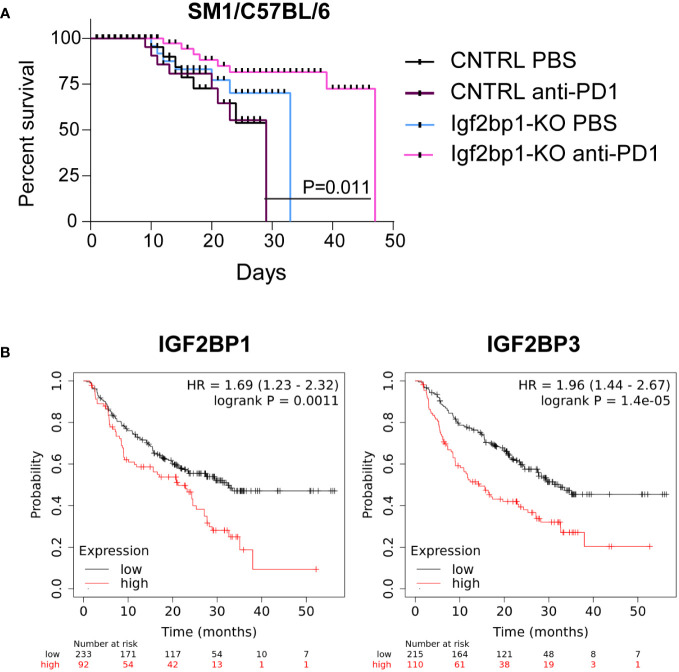
Downregulation of IGF2BP1 increases sensitivity to immunotherapy in mouse and human melanoma. **(A)** Kaplan-Meier survival analysis of C57BL/6 melanoma mouse model injected with 0.7x10^6^ SM1 cells carrying Igf2bp1-KO (*n*=20) or gRNA non-targeting CRISPR/Cas9 control (*n*=20), treated with either PBS (*n*=20) or anti-PD-1 antibodies (*n*=20); **(B)** a retrospective statistical analysis of human melanoma patient survival treated with immunotherapies, considering endogenous low or high levels of IGF2BP1 and IGF2BP3 expression (p<0.01, p<0.001 respectively).

Based on our observation that Igf2bp1 genetic inhibition in tumor cells increases expression of pro-inflammatory genes, increases sensitivity of melanoma cells to interferons stimulation, induces stimulation of intracellular Ifn-γ production in tumor-specific mouse T lymphocytes, and increases accumulation of immune cells in the tumor site, we performed *in vivo* modeling of anti-PD1 immunotherapy using SM1 mouse melanoma cells with CRISPR/Cas9 control and Igf2bp1-KO in syngeneic C57BL/6 mice. Mice were injected with SM1 CRISPR/Cas9 control (*n*=20) cells or SM1 Igf2bp1-KO (*n*=20) cells. Half of mice in each group (*n*=10) received anti-PD-1 antibodies or PBS twice a week. The analysis of tumor’s size revealed that SM1 Igf2bp1-KO tumors grew slower compared to SM1 CRISPR/Cas9 control. Anti-PD-1 treatment reduced the size of both CRISPR/Cas9 control and Igf2bp1-KO tumors, but the difference was not significant due to large variability in measurements (**Supplementary Figure 5A**). Similar, but not statistically significant, results were documented in a pilot experiment with combined anti-PD-1 antibody and IL-2 treatments in SW1 Igf2bp1-KD in C3H mice (**Supplementary Figure 5B**) However, we have observed a significant increase in mouse survival with anti-PD-1 treatment in larger groups (*n*=10 per group, *n*=40 total) when Igf2bp1-KO was combined with anti-PD1 antibody treatment compared to PBS control in C57BL/6 mouse model (**Figure 5A**).

To further validate our finding that high endogenous levels of IGF2BP proteins’ expression may have a suppressive effect on melanoma immunotherapy, we analyzed publicly available datasets of human melanoma patients outcomes, whose treatments include the administration of immunotherapy (37). We have found that patients with high expression levels of IGF2BP1 and IGF2BP3 have statistically significant poorer survival rates compared to those whose melanoma cells had low IGF2BP1 and IGF2BP3 levels (**Figure 5B**). These data indicate that expression levels of IGF2BPs may negatively correlate with sensitivity and success of immunotherapy in human melanoma patients and establish IGF2BPs as a possible target for future combinatory treatments.

## 4 Discussion

The oncofetal properties of the IGF2BP family of proteins, with a focus on their pro-proliferative effects, are well-documented (3). We and others demonstrated the role of IGF2BP proteins in maintaining levels of key regulators of cell cycle progression and self-renewal, and their cancer cell-autonomous immune-independent effects in tumor progression (4, 5, 21, 38–40). However, the role of IGF2BP1 and its paralogs in regulating Type I and II IFN signaling affecting innate and adaptive immune responses in cancer cells and outcomes of immunotherapies were not previously reported.

In this study, we demonstrate that genetic inhibition of the IGF2BP proteins in human and mouse cancer cells is associated with an increased expression of type I and/or II interferon-stimulated genes, and genes involved in cellular response to pathogens. The IGF2BP1, 2, and 3 loss-of-function systems in HEK293T cells showed that, among three paralogs, IGF2BP1 has a more notable effect on ISGs induction, with and without stimulation with cytokines. Upon IFN-beta treatment of IGF2BP1-deficient HEK293T cells, the expression of interferon beta- and gamma-regulated genes significantly increased, suggesting a possible role of this RBP in regulating a crosstalk between two signaling pathways. Further studies are required to understand how IGF2BPs convey innate immunity responses in mammalian cells. Known as multifunctional, single strand RNA-binding proteins, whose localization in a cytosol is associated with mRNA translation machinery and endoplasmic reticulum (41), IGF2BP may interact and inhibit various components of the nucleic acid sensor system.

We previously showed that IGF2BP1 stabilizes the ubiquitin ligase receptor β-TrCP1 mRNA and increases activity of SCF^β-TrCP1^ E3 ubiquitin ligase in response to activation of WNT/β-catenin signaling. We demonstrated a physiological significance of SCF^β-TrCP1^ E3 ubiquitin ligase’s protein substrates, β-catenin and the inhibitor of NF-kB (IKKb), for colon and melanoma cancer progression as high levels of human IGF2BP1 in these cancers were associated with NF-kB-activated antiapoptotic programs (40, 42). In 2004, Kumar et al, characterized the IFNAR1 subunit of Type I Interferon receptor as another important substrate of SCF^β-TrCP1^ E3 ubiquitin ligase (43). Therefore, one possible mechanism responsible for IGF2BP1-dependent regulation of Type I IFN signaling in mammalian cells is via β-TrCP1-dependent degradation of IFNAR1. Indeed, Ifnar1 knockout PVMM cells were significantly less sensitive (than the Ifnar1 WT SM1 cells) to the effects of Igf2bp1 downregulation on IFN signaling, suggesting that the Igf2bp1-βTrCP1-Ifnar1 pathway is at least partially responsible for the observed Igf2bp1-mediated phenotype. The capacity of PVMM Ifnar1^-/-^ cells to inhibit mrIfn-gamma response was rather unexpected, but consistent between independent biological replicates. This can be explained by a possible spontaneous mutation in the interferon gamma receptors, or an indirect effect of Ifnar1 gene knockout in these cells.

Two mouse melanoma models used in this study, Igf2bp1-KO in SM1/C57BL/6 and the doxycycline inducible Igf2bp1-KD in SW1/C3H, demonstrated the reduction of tumor’s size and the increased numbers of lymphoid and myeloid immune cells present in those tumors. The increased number of immune cells can be explained by the enhanced expression of pro-inflammatory genes and the increased presentation of mouse MHC I (H-2) complex on Igf2bp1-depleted melanoma cells. Indeed, mouse melanoma cells with Igf2bp1, 2 and 3 knockouts demonstrated the increased expression of H-2D^b^ and H-2K^b^ MHC I, enhancing a cell capacity of inducing intracellular Ifn-γ expression in syngeneic T-lymphocytes. A recent study by Liu et al. proposes allosteric regulation of IGF2BP as a novel strategy for the activation of tumor immune microenvironment in a hepatocellular carcinoma model (20). Liu et al., showed that chemical inhibition of RNA-binding KH-domain of IGF2BP1 reduced tumor size and increased infiltration of tumor site with immune cells, which is in line with our data.

Finally, our mouse melanoma model of anti-PD1 immunotherapies showed a significant increase of mouse survival when PD-1 inhibition is combined with inhibition of Igf2bp1. In our retrospective analysis of human melanoma patients who received immunotherapy, high levels of IGF2BP1 and IGF2BP3, but not IGF2BP2, were associated with poor clinical outcomes. This might be due to the fact that IGF2BP1 and IGF2BP3 are oncofetal proteins that are not expressed in most adult tissues but often reactivated in cancer, whereas IGF2BP2 expression is retained throughout adulthood. In addition, IGF2BP1 and IGF2BP3 are closely related structurally, sharing 73% of the amino acid sequence, while the overall amino acid sequence similarity between the three proteins is 56% (3). Several research groups conducted comprehensive studies dissecting the molecular mechanisms of immunotherapy resistance in melanoma patients with the focus on the role of neoepitopes and a gene expression signature of therapy resistance (44–46). Jerby-Arnon L. et. al., identified a resistance program associated with T cell exclusion and immune evasion (46). RNA sequencing analysis, conducted by Jerby-Arnon L. et. al., placed pro-proliferative IGF2BPs among differentially expressed genes of the immunotherapy responders versus non-responders, which are downregulated in “on-therapy” samples as well as some of their previously described mRNA targets e.g., *MITF*, *MYC*, *HOXB9*. Future studies will shed light on the mechanisms by which IGF2BPs alter interferon pathways and establish a possible application of IGF2BP inhibitors alongside immunotherapies in human cancers.

## Data availability statement

The original contributions presented in the study are publicly available. This data can be found here: GEO database, accession number GSE236818.

## Ethics statement

The animal study was reviewed and approved by Penn State Health and College of Medicine Research Quality Assurance (RQA), IACUC Office of the Vice Dean for Research and Graduate Studies.

## Author contributions

IE, TS, and VS developed a concept, designed and performed experiments, and analyzed data. IE made figures and wrote the paper. CG performed and analyzed *in vivo* and *in vitro* experiments. DB, SG, AF, NS, and KK performed experiments and analyzed data. AS and SD analyzed and interpreted data. YU performed bioinformatics analysis and made figures. All authors contributed to manuscript editing. All authors contributed to the article and approved the submitted version.
