# Erratum: IGF2BP family of RNA-binding proteins regulate innate and adaptive immune responses in cancer cells and tumor microenvironment

**DOI:** 10.3389/fimmu.2023.1297519

**Published:** 2023-09-27

**Authors:** Frontiers Production Office

**Affiliations:** Frontiers Media SA, Lausanne, Switzerland

**Keywords:** IGF2BP/IMP, RNA-binding protein (RBP), interferon-stimulated genes (ISGs), melanoma, leukemia, anti-PD-1, immunotherapy, cancer stem cell (CSC)

Due to a production error, an incorrect Data Availability statement was provided. The correct Data Availability statement appears below.

“The original contributions presented in the study are publicly available. This data can be found here: GEO database, accession number GSE236818”.

The publisher apologizes for this mistake.

The original version of this article has been updated.

