# Supplementary material for: IGF2BP family of RNA-binding proteins regulate innate and adaptive immune responses in cancer cells and tumor microenvironment
Source: Front Immunol. 2023 Jul 12;14:1224516. doi: 10.3389/fimmu.2023.1224516 (PMC10369348; doi:10.3389/fimmu.2023.1224516)
Supplement: Supplementary file 1 [file DataSheet_1.pdf]

Supplementary Material

IGF2BP family of RNA-binding proteins regulate innate and adaptive immune responses in cancer cells and tumor microenvironment

Irina A. Elcheva[1]*, Chethana P. Gowda[1], Daniel Bogush[1], Svetlana Gornostaeva[1], Anna Fakhardo[1], Neil Sheth[1†], Kathleen M. Kokolus[2†], Arati Sharma[3], Sinisa Dovat[1], Yasin Uzun[1,4], Todd D. Schell[2] and Vladimir S. Spiegelman[1]*

*** Correspondence:** Irina A. Elcheva, PhD: ielcheva@pennstatehealth.psu.edu; Vladimir S. Spiegelman, MD, PhD: vspiegelman@pennstatehealth.psu.edu.

1. Supplementary Data

1.1 Supplementary Data Table 1.1 B-ALL 697 (EU3) upregulated ISG (supplementary to Figure 1A, GO and KEGG pathways analysis by STRING software).

Gene ID	Gene Symbol	Fold	log2(fold)	P_values	FDR
**ENSG00000130303**	BST2	1.4233	0.50921	0.035205	0.25424
**ENSG00000162645**	GBP2	1.5429	0.62568	0.038751	0.26595
**ENSG00000206505**	HLA-A	1.7071	0.77156	0.001252	0.038145
**ENSG00000225691**	HLA-C	2.0896	1.0632	1.76E-05	0.00206
**ENSG00000137965**	IFI44	25.439	4.669	1.54E-06	0.000313
**ENSG00000126709**	IFI6	5.5252	2.466	6.99E-17	2.36E-13
**ENSG00000119922**	IFIT2	2.6329	1.3966	0.0044969	0.083919
**ENSG00000119917**	IFIT3	3.5156	1.8138	3.00E-08	1.26E-05
**ENSG00000128604**	IRF5	1.794	0.84317	0.0067565	0.10432
**ENSG00000259529**	IRF9	2.3325	1.2219	0.0066862	0.1037
**ENSG00000187608**	ISG15	2.9979	1.5839	6.89E-10	5.65E-07
**ENSG00000172183**	ISG20	1.8282	0.87046	0.00042836	0.018179
**ENSG00000157601**	MX1	4.9881	2.3185	3.00E-15	7.22E-12
**ENSG00000111335**	OAS2	1.63	0.70491	0.008735	0.11878
**ENSG00000111331**	OAS3	3.0504	1.609	0.00015699	0.009153
**ENSG00000135114**	OASL	20.544	4.3606	5.50E-07	0.000136
**ENSG00000235715**	PSMB8	7.5363	2.9139	0.045297	0.28404
**ENSG00000101347**	SAMHD1	5.2889	2.403	1.59E-14	3.34E-11
**ENSG00000115415**	STAT1	1.4474	0.53346	0.037127	0.26063

1.2 Supplementary Figures

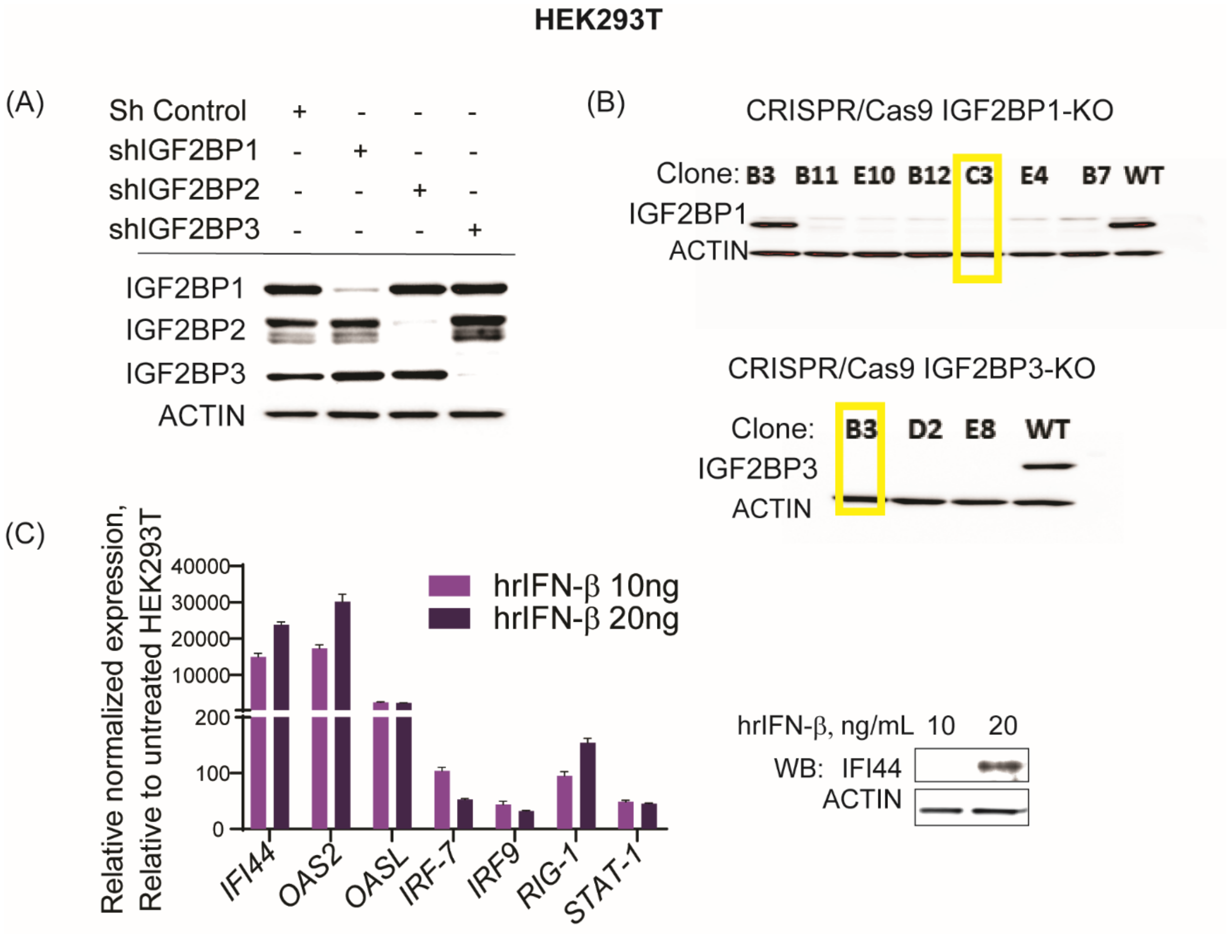

Supplementary Figure 1. IGF2BP1-3 loss-of-function systems and hrIFN-β treatment validation in HEK293T cells. **(A)** western blot analysis of IGF2BP1, 2, and 3 expression in HEK293T cells expressing shRNA against IGF2BP paralogs or non-targeting shControl; **(B)** western blot analysis of CRISPR/Cas9 IGF2BP1, 3 gene knockout clones (examples of western blotting for some of the clones used in this study are framed); **(C)** hrIFN-β treatment validation by qPCR, western blotting for IFI44 (10, 20 ng/mL, 12 hrs.).

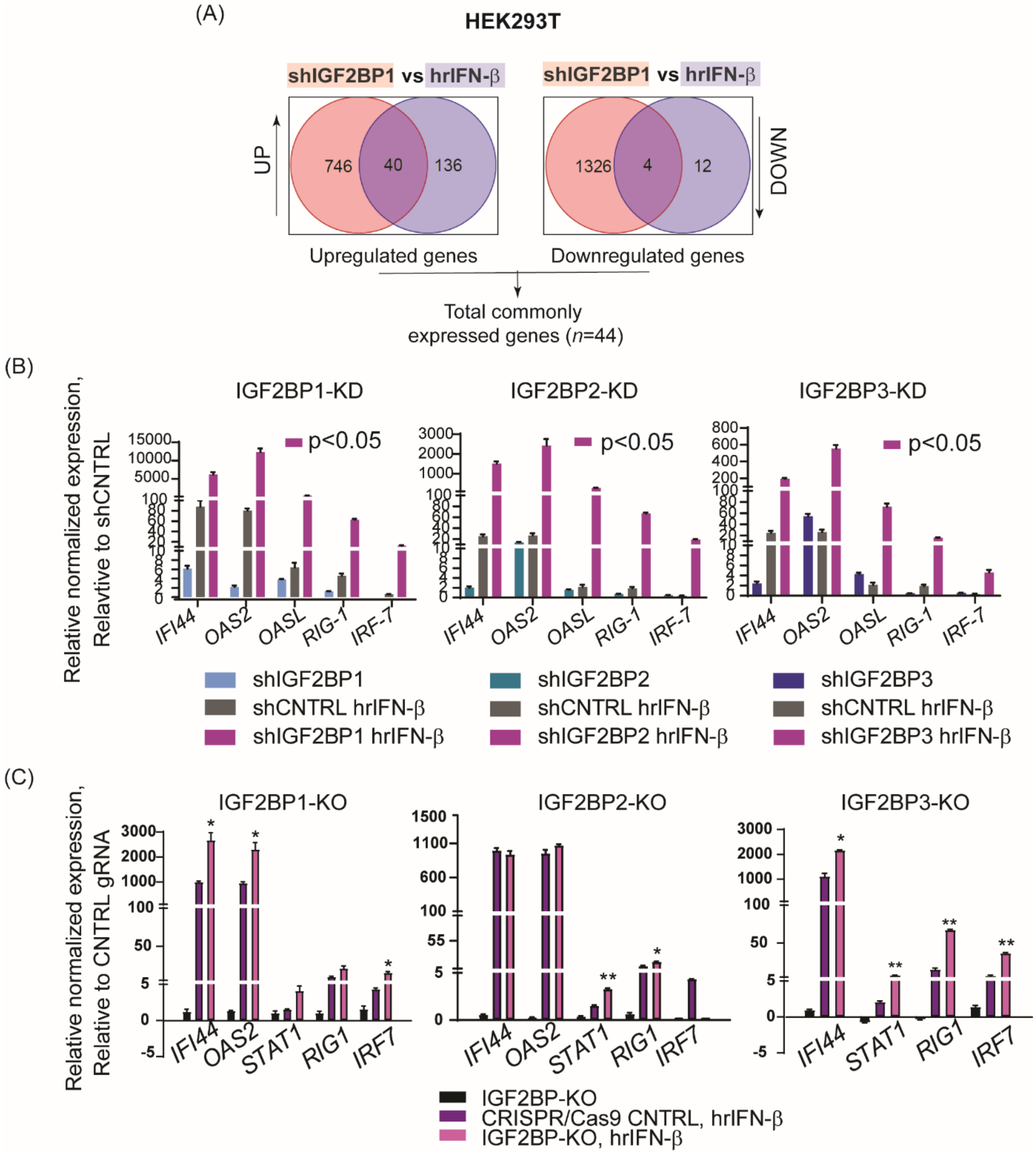

Supplementary Figure 2. IGF2BPs regulate type – I and type-II IFN-stimulated gene expression in mammalian cells. (A) HEK293T cells mRNA-seq analysis: VENN diagrams of differentially and commonly expressed genes in HEK293T cells with shIGF2BP1 gene knockdown versus HEK293T cells treated with human recombinant (hr) IFN-β; **(B)** expression levels of innate immunity genes assessed by qPCR in HEK293T cells unstimulated or stimulated with hrIFN- β (10ng/mL, 12 hours), transduced with pLKO.puro vectors expressing non-targeting shControl and shRNA against IGF2BP1-3 (KD), or **(C)** CRISPR/Cas9 IGF2BP1-3 gene knockouts (KO), relative to non-targeted, unstimulated controls.

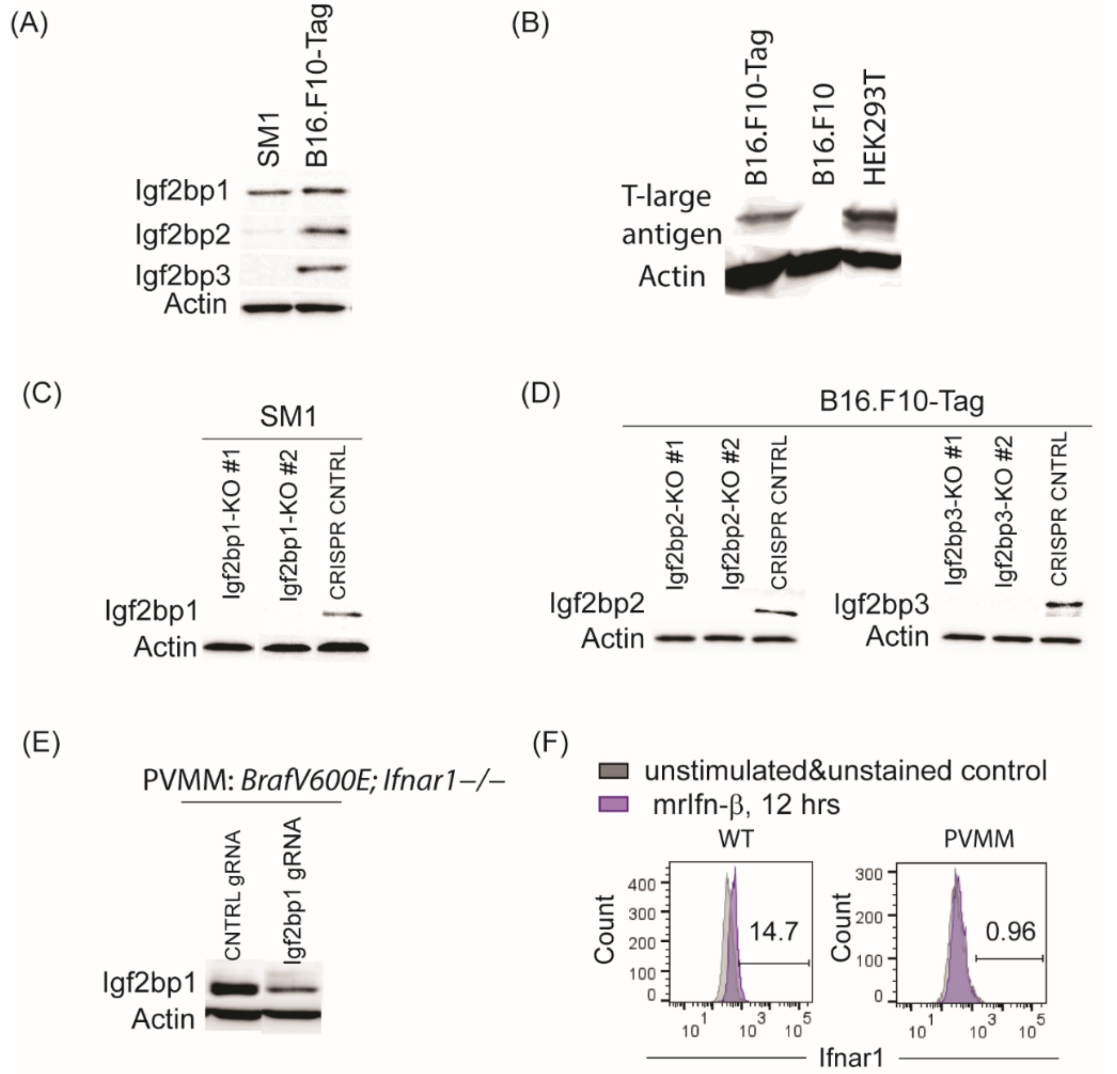

Supplementary Figure 3. Igf2bp1-3 loss-of-function systems in mouse melanoma cells. (A) western blot analysis of endogenous Igf2bp1, 2, and 3 expression in mouse melanoma SM1 and B16.F10-Tag cells; **(B)** western blot analysis of large T antigen in B16.F10-Tag, B16.F10 (parental, negative control), and HEK293T (positive control); **(C)** western blot analysis of endogenous Igf2bp1 expression in mouse melanoma SM1 CRISPR/Cas9 non-targeting control and Igf2bp1-KO clones; **(D)** western blot analysis of endogenous Igf2bp2, and 3 expression in B16.F10-Tag cells CRISPR/Cas9 control, Igf2bp2-KO, and Igf2bp3-KO clones**; (E)** western blot analysis of endogenous Igf2bp1 expression in mouse melanoma PVMM (BrafV600E; Ifnar1-/-), CRISPR/Cas9 non-targeting or Igf2bp1 gRNA-modified cells; **(F)** flow cytometric analysis of mouse Ifnar1 expression in PVMM (BrafV600E; Ifnar1-/-) or wild type mouse melanoma cells before (unstimulated and unstained controls (grey), and after mrIfn-β stimulation (1000U/mL, 12 hours); wild type Ifnar1 (14.7%), PVMM (0.96%).

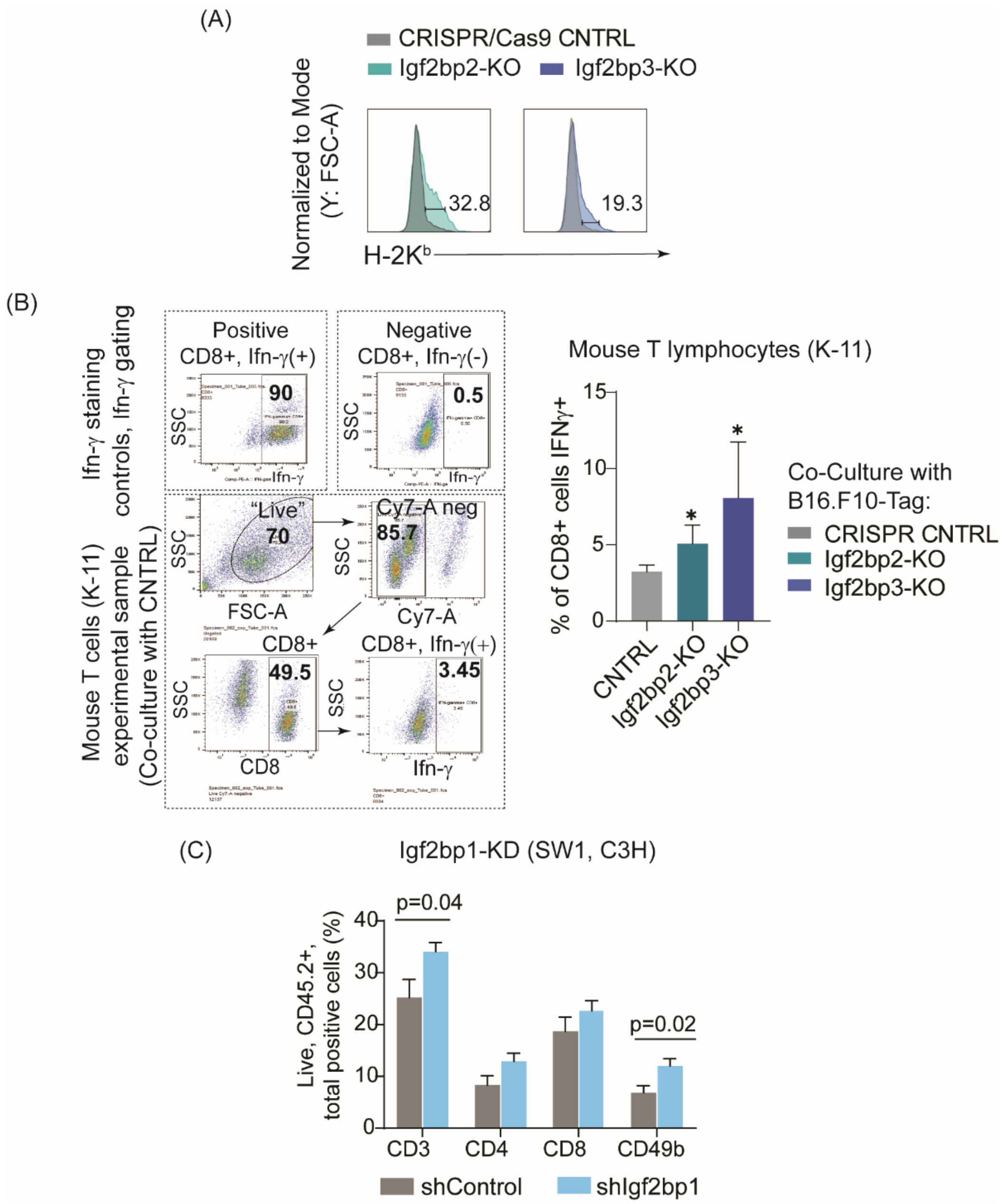

Supplementary Figure 4. Downregulation of Igf2bp family of proteins increases immunogenicity of mouse melanoma cells and inflames tumor microenvironment. (A) flow cytometric analysis of mouse H-2K^{b} surface expression on B16.F10-Tag Igf2bp2, 3-KO cells compared to CRISPR/Cas9 control; **(B)** gating strategy of intracellular Ifn-γ expression in live, CD8+ K-11 T-cells (sample), positive (Tag206-215 peptide stimulation) and negative (no stimulation) Ifn-γ staining controls, and quantification of Ifn-γ staining in Igf2bp2, 3-KO, CRISPR/Cas9 controls clones (*n*=4); **(C)** flow cytometric analysis of mouse tumor microenvironment in doxycycline-inducible Igf2bp1-KD compared to shControl tumors in syngeneic C3H mice (*n*=7), number positive (%) T-cells (CD3, CD4, CD8), Natural Killer (NK) cells (CD49b) in mouse melanoma tumors generated by subcutaneous injection of 0.5x10^{6} SW1 cells.

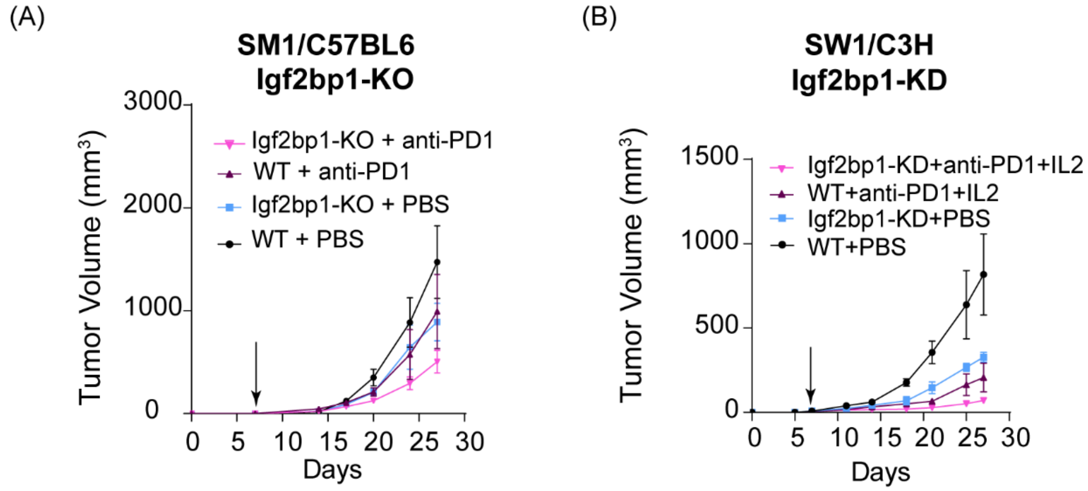

Supplemental Figure 5. Downregulation of Igf2bp1 increases sensitivity to immunotherapy in mouse melanoma. (A) Tumor volume measurements for SM1 C57BL/6 melanoma mouse model using CRISPR/Cas9-induced Igf2bp1-KO and gRNA non-targeted CRISPR/Cas9 Control (WT on the graph), treated with PBS, or anti-PD-1 antibodies (*n*=10 for each group, *n*=40 total); **(B)** Tumor volume measurements in SW1/C3H melanoma mouse model using Igf2bp1-KD and non-targeting shRNA Control (WT), treated with PBS, or combined anti-PD-1 antibody and IL-2 (*n*=5); Arrow indicates day 7 post-injection, when tumor volume measurement begun.

2 Supplementary Materials and Methods

2.1 Table 2.1. Expression plasmids and transfection reagents

Construct/Plasmid Name	Company	Catalog #	Sequence/ Gene ID
**UltraCruz® Transfection Reagent**	Santa Cruz Biotechnology	sc-395739 UltraCruz® Transfection Reagent	n/a
**Control CRISPR/Cas9 Plasmid**	Santa Cruz Biotechnology	sc-418922	n/a
**IGF2BP1 CRISPR/Cas9 KO Plasmid (mouse)**	Santa Cruz Biotechnology	sc-431212	140486
**IGF2BP2 CRISPR/Cas9 KO Plasmid (mouse)**	Santa Cruz Biotechnology	sc-435664	319765
**IGF2BP3 CRISPR/Cas9 KO Plasmid (mouse)**	Santa Cruz Biotechnology	sc-431213	140488
**IGF2BP1 CRISPR/Cas9 KO Plasmid (human)**	Santa Cruz Biotechnology	sc-401703	10642
**IGF2BP2 CRISPR/Cas9 KO Plasmid (human)**	Santa Cruz Biotechnology	sc-404985	10644
**IGF2BP3 CRISPR/Cas9 KO Plasmid (human)**	Santa Cruz Biotechnology	sc-402603	10643
**shIGF2BP1/IMP1 MISSION® pLKO.1-puro**	Sigma	TRCN0000218079	ACGCTTAGAGATTGAACATTC (Gene ID: 10642)
**shIGF2BP2/IMP2 MISSION® pLKO.1-puro**	Sigma	TRCN0000255463	GGTGCCTGCAGCGGTAATATA (Gene ID: 10644)
**shIGF2BP3/IMP3 MISSION® pLKO.1-puro**	Sigma	TRCN0000074675	CGGTGAATGAACTTCAGAATT (Gene ID: 10643)
**shControl MISSION® pLKO.1-puro**	Sigma	SHC002	Targets no known mammalian genes
**shRNA SMARTvector Inducible Non-targeting Control construct**	GE Dharmacon	VSC11654	Targets no known mammalian genes
**Sh IGF2BP1 (mouse) SMARTvector Inducible TurboGFP shRNA Lentiviral constructs**	GE Dharmacon	V3SM11256-03EG140486	140486

2.2 Table 2.2 PrimePCR™ SYBR® Green Assay (validated DNA primers qPCR from BioRad).

Gene Name	Bio-Rad ID
**Ifnb**	qMmuCED0050444
**Ifng**	mMmuCID0006268
**IFNG**	qHsaCID0017614
**IFNB1**	qHsaCED0019234
**IGF2BP1**	qHsaCID0010743
**IGF2BP2**	qHsaCED0036334
**IGF2BP3**	qHsaCED0004904
**Rps18**	qMmuCED0045430
**RPS18**	qHsaCED0037454

2.3 Table 2.3 Other primers used in this study.

Gene Name	Fwd	Rev
**Ddx58**	CAGATCCGAGACACTAAAGGGA	TCCTCATCAGCCTTGCTTTCA
**Ifi44**	AACTGACTGCTCGCAATAATGT	GTAACACAGCAATGCCTCTTGT
**Mavs**	CTGCCTCACAGCTAGTGACC	CCGGCGCTGGAGATTATTG
**Stat1**	TCACAGTGGTTCGAGCTTCAG	GCAAACGAGACATCATAGGCA
**Oas2**	TTGAAGAGGAATACATGCGGAAG	GGGTCTGCATTACTGGCACTT
**Irf9**	GCCGAGTGGTGGGTAAGAC	GCAAAGGCGCTGAACAAAGAG
**Irf7**	GAGACTGGCTATTGGGGGAG	GACCGAAATGCTTCCAGGG
**RIG-1**	TGCGAATCAGATCCCAGTGTA	TGCCTGTAACTCTATACCCATGT
**OASL**	CTGATGCAGGAACTGTATAGCAC	CACAGCGTCTAGCACCTCTT
**IRF-9**	GCCCTACAAGGTGTATCAGTTG	TGCTGTCGCTTTGATGGTACT
**IFI44**	GGTGGGCACTAATACAACTGG	CACACAGAATAAACGGCAGGTA
**MAVS**	TTCTAATGCGCTCACCAATCC	CCATGCTAGTAGGCACTTTGGA
**IRF-7**	CCCAGCAGGTAGCATTCCC	GCAGCAGTTCCTCCGTGTAG
**STAT1**	CGGCTGAATTTCGGCACCT	CAGTAACGATGAGAGGACCCT
**OAS2**	ACGTGACATCCTCGATAAAACTG	GAACCCATCAAGGGACTTCTG
**RPL13A #1**	CCTGGAGGAGAAGAGGAAAGAGA	TTGAGGACCTCTGTGTATTTGTCAA
**RPL13A #2**	CGGACCGTGCGAGGTAT	CACCATCCGCTTTTTCTTGTC
**RPS18**	ATCACCATTATGCAGAATCCACG	GACCTGGCTGTATTTTCCATCC
**TBP**	CCCGAAACGCCGAATATAATCC	AATCAGTGCCGTGGTTCGTG

2.4 Table 2.4. Antibodies

Gene Name	Company	Catalog #	Clone	IgG Type	Dilution
**Fixable Viability stain 780**	BD	BDB565388	n/a	n/a	
**CD45.2BUV395**	BD	564616	Clone 104 (RUO)	Mouse SJL IgG2a, κ	1:100
**CD3e BB700**	BD	566494	145-2C11 (RUO)	Armenian Hamster IgG1, κ	1:100
**CD8a BV786**	BD	563332	53-6.7 (RUO)	Rat LOU (Louvain, LOU/C, LOU/M IgG2a, κ)	1:100
**CD4 BUV737**	BD	612844	RM4-5 (RM4.5) (RUO)	Rat DA, DA/HA IgG2a, κ	1:100
**CD19 APC**	BD	550992	1D3 (RUO)	Rat LEW, Lewis IgG2a, κ	1:100
**NK1.1 BB700**	BD	562921	PK136 (RUO)	Mouse C3H x BALB/c IgG2a, κ	1:100
**CD49b BV421**	BD	563063	DX5	Rat/IgM, κ	1:100
**CD19 BB700**	BD	566411	1D3 (RUO)	Rat LEW, also known as Lewis IgG2a, κ	1:100
**NK1.1**	BD	566502	PK136 (RUO)	Mouse C3H x BALB/c IgG2a, κ	1:100
**CD11b BV605**	BD	563015	PK136 (RUO)	Mouse C3H x BALB/c IgG2a, κ	1:100
**CD11c BUV737**	BD	749039	N418 (RUO)	Armenian Hamster IgG2	1:100
**F4/80 BV711**	BD	565612	T45-2342 (RUO)	Rat WI (Wistar (outbred) IgG2a, κ)	1:100
**Ly-6C APC**	BD	560595	AL-21 (RUO)	Rat IgM, κ	1:100
**LY-6G BV421**	BD	562737	1A8 (RUO)	Rat LEW (Lewis IgG2a, κ)	1:100
**IFNgamma-FITC**	Tonbo Biosciences	35-7311-U100	XMG1.2	Rat/IgG1, κ	1:100
**H-2Kb**	ThermoFisher Sigma	Y200-1MG MABF946	Y-3	Mouse/IgG2b,k	1:100
**H-2Db**	ThermoFisher	MA5-17992	B22.249	Mouse/IgG2a	1:100
**beta-Actin**	Cell Signaling Technologies	4970	13E5	Rabbit IgG	1:2000
**HRP-linked anti-rabbit**	Cell Signaling Technologies	7074	n/a	Goat	1:2000
**IGF2BP1**	MBL	RN007P	n/a	Rabbit IgG	1:1000
**IGF2BP1**	Cell Signaling Technologies	8482	D33A2	Rabbit IgG	1:1000
**IGF2BP2**	MBL	RN008P	n/a	Rabbit IgG	1:1000
**IGF2BP3**	MBL	RN009P	n/a	Rabbit IgG	1:1000
**Anti-mouse CD279 (PD-1)**	Leinco Technologies, Inc.	P372-25 mg	RMP1-14	Rat / IgG2a κ	200µg per treatment in vivo
